# Approaches for enhancing the informativeness and quality of clinical trials: Innovations and principles for implementing multicenter trials from the Trial Innovation Network

**DOI:** 10.1017/cts.2023.560

**Published:** 2023-05-25

**Authors:** Karen Lane, Marisha E. Palm, Eve Marion, Marie T. Kay, Dixie Thompson, Mary Stroud, Helen Boyle, Shannon Hillery, Angeline Nanni, Meghan Hildreth, Sarah Nelson, Jeri S. Burr, Terri Edwards, Lori Poole, Salina P. Waddy, Sarah E. Dunsmore, Paul Harris, Consuelo Wilkins, Gordon R. Bernard, J. Michael Dean, Jamie Dwyer, Daniel K. Benjamin, Harry P. Selker, Daniel F. Hanley, Daniel E. Ford

**Affiliations:** 1 Department of Neurology, Johns Hopkins University School of Medicine, Baltimore, MD, USA; 2 Tufts Medical Center, Boston, MA, USA; 3 Institute for Clinical Research and Health Policy Studies, Tufts Medical Center, Boston, MA, USA; 4 Duke Clinical Research Institute, Duke University School of Medicine, Durham, NC, USA; 5 University of Utah School of Medicine, Salt Lake City, UT, USA; 6 Vanderbilt Institute for Clinical and Translational Research, Vanderbilt University Medical Center, Nashville, TN, USA; 7 Johns Hopkins University School of Medicine, Baltimore, MD, USA; 8 Vanderbilt University Medical Center, Nashville, TN, USA; 9 Division of Clinical Innovation, National Center for Advancing Translational Sciences, Bethesda, MD, USA; 10 Vanderbilt School of Medicine, Nashville, TN, USA; 11 Department of Medicine, Vanderbilt University Medical Center, Nashville, TN, USA; 12 Tufts Clinical and Translational Science Institute, Tufts University, Boston, MA, USA; 13 Health Policy Studies, Tufts Medical Center, Boston, MA, USA; 14 Acute Care Neurology, Johns Hopkins University School of Medicine, Baltimore, MD, USA; 15 Institute for Clinical and Translational Research, Johns Hopkins University School of Medicine, Baltimore, MD, USA

**Keywords:** Multicenter trials, informative trials, Trial Innovation Network, study planning, clinical trial budgets, clinical trial resources

## Abstract

One challenge for multisite clinical trials is ensuring that the conditions of an informative trial are incorporated into all aspects of trial planning and execution. The multicenter model can provide the potential for a more informative environment, but it can also place a trial at risk of becoming uninformative due to lack of rigor, quality control, or effective recruitment, resulting in premature discontinuation and/or non-publication. Key factors that support informativeness are having the right team and resources during study planning and implementation and adequate funding to support performance activities. This communication draws on the experience of the National Center for Advancing Translational Science (NCATS) Trial Innovation Network (TIN) to develop approaches for enhancing the informativeness of clinical trials. We distilled this information into three principles: (1) assemble a diverse team, (2) leverage existing processes and systems, and (3) carefully consider budgets and contracts. The TIN, comprised of NCATS, three Trial Innovation Centers, a Recruitment Innovation Center, and 60+ CTSA Program hubs, provides resources to investigators who are proposing multicenter collaborations. In addition to sharing principles that support the informativeness of clinical trials, we highlight TIN-developed resources relevant for multicenter trial initiation and conduct.

## Introduction

Multicenter clinical trials provide greater evidence on the generalizability of an intervention than single center trials. Other strengths include a larger sample size, conduct across different settings, greater diversity in participants, the development of protocols that receive input from multiple research teams, and ethical scrutiny from a wider variety of institutions, thereby protecting both participants and public health.

Multicenter studies provide external validation of the protocol, equipoise in more than one clinical setting, and added insights into a trial’s value and impact, and they naturally promote a broader review of the trial protocol and the identification of possible critical design or operational details that could render the study more informative [1]. However, these strengths are accompanied by great challenges: multicenter trials are more complex, expensive, and time consuming and require more intense review before funding and greater teamwork with coinvestigators during study training and start-up.

Insufficient responses to challenges and training/planning needs can place a multicenter trial at risk of becoming uninformative, particularly if it is unable to identify and retain active, enthusiastic centers or complete in a timely manner due to insufficient funding. These challenges often lead to premature study closure and the inability to publish meaningful trial results [2]. In addition to ensuring that a study is asking an important and unresolved scientific question and is credibly designed, controlling conditions that support feasibility, trial conduct, and trial reporting becomes harder as research is scaled-up across diverse centers. The handoff of protocol activities to centers where new investigative teams, less familiar with the protocol design, need training and quality controls to ensure balanced enrollment, and adequate data quality is complex. Important to the informativeness and quality of a multicenter clinical trial is the ability to operationalize a coalition of teams and centers in ways that the informative conditions of the tightly knit single center – uniformity, homogeneity, local quality control, and performance visibility – continue to be met.

## Background

Inefficiencies in multicenter clinical research infrastructure have been apparent for decades [1]. In 2016, The NIH National Center for Advancing Translational Science (NCATS) formed the Trial Innovation Network (TIN) to increase efficiency and effectiveness of multicenter clinical trials by focusing on innovation and collaboration and by leveraging the strength and expertise of the Clinical and Translational Science Award (CTSA) Program. The TIN is comprised of four key partnerships: NCATS, CTSA Program hubs, three Trial Innovation Centers (TICs), and a Recruitment Innovation Center (RIC) [3].

The NCATS TIN team is part of the Clinical Affairs Branch of the Division of Clinical Innovation which plans, conducts, and supports research across the clinical phases of the translational science spectrum and oversees the Clinical and Translational Science Award (CTSA) Program. The NCATS team is led by the Clinical Affairs Branch Chief who also serves as Director of the TIN and includes a team of program directors, clinical operations, and support staff. NCATS staff members fully participate in TIN working groups, and TIC and RIC program directors work as project collaborators on all operational Trial Innovation Network projects.

The CTSA Program hubs are a network of 65+ medical research institutions dedicated to improving the efficiency, quality, and impact of translational research [4]. Specific to multicenter clinical trials, CTSA Program-wide efforts have included recruitment strategizing, good clinical practice qualification and competency training, feasibility assessments, risk management, and Institutional Review Board (IRB) and contract streamlining.

The TICs and RIC work together through the TIN proposal process to provide planning guidance and operational assistance to investigative teams wishing to lead multicenter clinical trials. The CTSA Program hubs work with the TICs and RIC to provide complementary institutional expertise and to identify sites and site investigators for multicenter studies. When feasible the impact of recruitment tools and operational innovations developed by the RIC and TICs is tested in multicenter trials that receive operational assistance from the TIN during the study startup and enrollment/retention phases of the trial [3].

**Figure 1.**
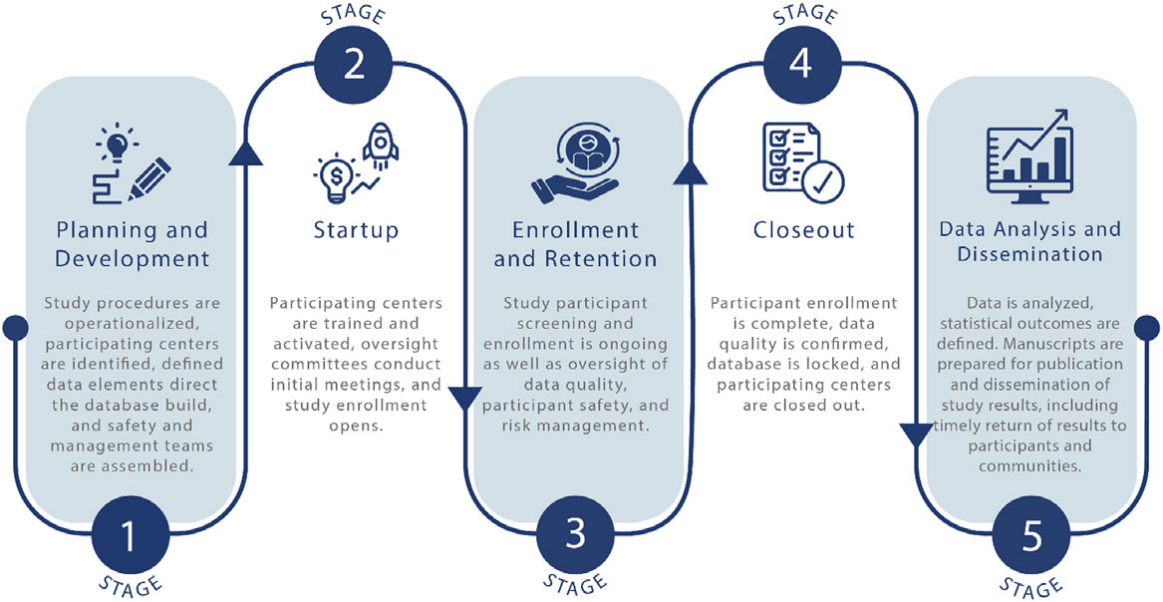
The multicenter trial life cycle can be divided into five stages. When scaling up from a single center trial, planners in stage one must operationalize the study for multiple settings and a more diversified participant population. To assure informativeness, the startup second stage requires more effort directed to training and quality assurance oversight to ensure teams provide an intervention consistently during stage three. The rewards are realized in stages four and five, when data quality is confirmed, and the results of the trial inform medical practitioners, participants, and communities.

A challenge brought to the TIN by CTSA hub leaders and staff has been how to transition from small, single center trials to multicenter trials. The multicenter trial life cycle can be divided into five stages (Fig. 1). A TIN working group met to identify the differences between single and multicenter trials at each stage of the trial cycle. Given the many details and nuances in scaling up to a multicenter study, the group considered potential ways to disseminate this information broadly. To support the scope of preparation and breadth of decisions that investigative teams must make, the group drafted key principles with accompanying tactics that support the planning and conduct of a successful multicenter trial. Embedded in these key principles are strategies that improve the ability of a trial to informatively guide clinical, policy, and research decisions [5,6].

We provide information on three principles that enhance the informativeness of multicenter trials. For each principle, we describe its connection to informativeness and provide a broader description of its importance and tactics that can support investigative teams, including the use of TIN developed tools. The principles are (1) assemble a diverse team, (2) leverage existing processes and systems, and (3) carefully consider budgets and contracts.

## Principle 1: Assemble a Diverse Team

### Factor That May Affect Informativeness

Multicenter investigators who do not have robust teams with diverse expertise and clear communication channels may struggle to operationalize the study, causing delays, suboptimal trial conduct, and uninformative results.

### Informed Approach

*Work with a trial management team and an executive committee (EC) from the start and convene special oversite committees (single Institutional Review Board, Data Safety Monitoring Board, Community Advisors) as soon as possible. Obtain commitments across stakeholders and select and train teams with strong incentives to conduct the research and who will follow and identify the critical operational details that make sure the trial results will be informative.*

### Description

Large, multicenter trials have the challenges of building an organization and planning the staffing and division of labor that go along with it. Although a single principal investigator (PI) can sometimes manage a small multicenter trial alone, large trials can benefit from additional co-investigators and teams, including statisticians, project managers, and center directors and managers, to work under the leadership of the study PI.

**Figure 2.**
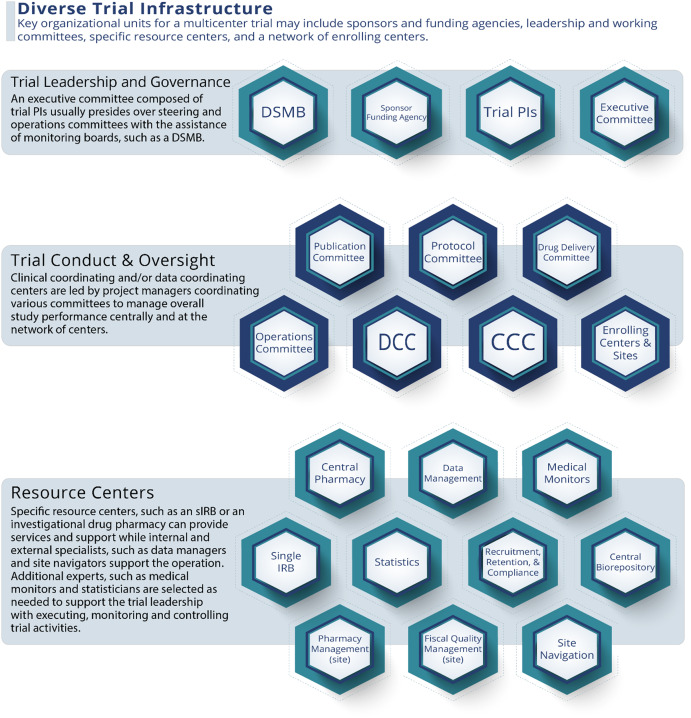
A sample organizational structure that outlines how a framework of essential management units and roles interact to support multicenter trial activities and enhance the informativeness and quality of a clinical trial.

Large multicenter trials require a diverse team or group of teams with multiple types of expertise. The harmonization of such a large, diverse team is central to the success of a study. Investigators must build a network of resource centers, common treatment and data collection protocols, and a coordinating center to receive and process study data [7]. The consortium expands even more with the addition of funding agency officers, internal and external committees, and vendors (e.g., central biorepositories, drug suppliers, and central pharmacies) (Fig. 2). It is this diverse consortium infrastructure, working together in trust and partnership, that becomes the driver of progress.

Actively engaged committees can be an excellent resource for accomplishing diversity and supporting the multiple types of expertise necessary to drive progress, nurture partnerships, and advance informativeness. An executive committee (EC) will usually lead and govern a multicenter trial and include a study chair, a project manager, statisticians, a few members from the enrolling centers, project officers, and coordinating center directors who, as a group, take on everyday decision-making. Under the guidance of the EC, multiple subcommittees, both standing and ad hoc, and with members representing each of the oversight and resource centers, are formed to oversee defined tasks.

The EC needs an efficient operations team, led by a project manager, to maintain high performance throughout the trial. Dedicated project managers have been shown to be a common component of successfully enrolling multicenter trials [9]. Project managers are responsible for mapping and communicating the processes of initiating, planning, executing, monitoring, and controlling trial activities and reporting on trial activities to committees, external teams and consultants, and data and safety monitoring boards. As effective operations team performance is essential to trial success, the project manager also assures that the operations team is cohesive and knows the study protocol, knows the tasks of other team members, and is familiar with project timelines and milestones. Cohesion across the study occurs when communication and training are informative and repetitive across roles and when operational procedures, organizational structures, and communication channels are codified early into a written communications plan [8].

Teams benefit from a diversity of ideas and perspectives. Researchers who have modeled team member selection across diverse groups of individuals found that groups comprised of diverse problem solvers outperformed teams made up of those deemed to be the best performing experts [9]. Once a diverse central team is assembled and the planning stage is complete, the second stage of the trial cycle provides the opportunity to build collaboration and commitment among a network of diverse enrollment centers (see Fig. 1). Investigators should emphasize that a trial is owned by a team, not an individual [10] and that a diversity of perspectives is embraced to guide decisions that improve trial outcomes and produce more informative research. As centers are brought on board, center teams are engaged in the common purpose and become unified in their understanding of why the research is important and the necessary commitments and performance expectations. Blending diversity and collective ownership can foster productivity, innovation, and better trial outcomes. It will also build trust in the value of the research and reduce the risk of bias among center teams, both keys to promoting informative trials [11,12].

### Principle 1 at Work in the Trial Innovation Network

In a recently funded trial in which a TIC assisted with study planning, six teams were created to cover six “planning zones” needed to launch the trial at multiple CTSA centers. The zones were: analysis and reporting, information technology, data development, operations, and clinical affairs, scientific and protocol, and financial operations. Instead of depending on a single team to do all the planning and building, the six teams were assigned study planning milestones with a specific ordering of tasks so milestones could be achieved on pre-specified delivery dates. Teams met weekly and reported to the PI and project manager at joint meetings at the end of each week.

As another resource, the TIN Toolbox, a repository of published guidelines and templated materials from across the CTSA consortium, contains many tools and documents relevant to study initiation, teamwork, and communication in all stages of multicenter clinical trials. The TIN Toolbox and other TIN resources can be found on its publicly accessible TIN website [13].

## Principle 2: Leverage Existing Processes and Systems

### Factor That May Affect Informativeness

Multicenter trialists may be unaware of institutional and virtual resources and partnerships available for guidance and replication, which could lead to inefficiencies.

### Informed Approach

*Work with institutional research navigators and administrators at the local CTSA hub level, involve NIH program officers in discussions, review sponsor websites; and seek special trial planning and consortia advisors, such as the TIN, as early organizational collaborators.*

### Description

An informed approach to trial planning involves reliable and rapid access to relevant expertise and published standards for trial management and conduct, yet trialists continue to reinvent the wheel [14]. As stated in a recent NCATS solicitation for TIN grant applications: “large multicenter clinical studies supported by both public and private sectors are often *sui generis*, with systems or processes developed from scratch and ignored after trial completion, despite their potential utility for future studies.”

Investigators organizing a multicenter trial should leverage existing governance structures and systems including federal, institutional, and specialized mentoring programs. On the federal level, NIH websites post an abundance of downloadable plans, schematics, guidelines, and templates; and NIH mentors include program and scientific officers who are available prior to grant submissions and post award, as are financial officers once a grant is funded. In addition, CTSA Program hub liaison teams across the US can serve as introductory points to TIN resources, as well as hub-specific resources such as Biostatistics, Epidemiology and Research Design units [15]. And turnkey software and computer systems are available to automate some trial tasks and provide efficient and more informed trial management. Proven, well-used processes and systems are likely to be accepted across multiple sites in a timely manner. An important consideration when choosing *new* processes and systems, such as EMR systems or other marketed software, is that implementation timelines may vary from institution to institution and markedly delay approval times.

### Principle 2 at Work in the Trial Innovation Network

**Figure 3.**
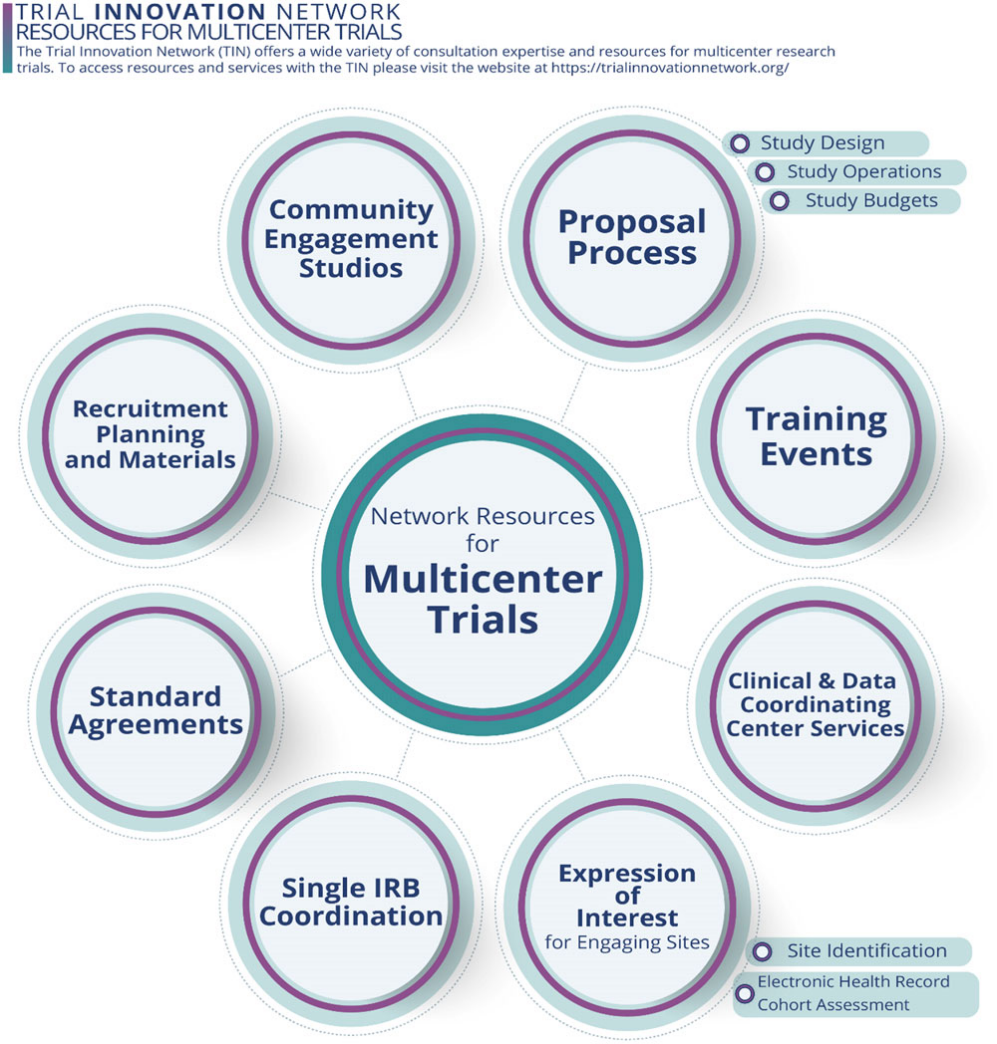
The Trial Innovation Network (TIN) offers a variety of network resources throughout the planning and implementation phases of multicenter clinical trials, each providing opportunities to further the informativeness of trial results and impact.

The TIN offers a variety of resources for multicenter trials (Fig. 3). In its first six years, the TIN has conducted 83 consultations for investigative teams of varying experience, including 32 first-time multicenter investigators. The TIN has used the lessons learned from these consultations to develop training events and materials that address the needs of multicenter investigators. The TIN also partners with investigators who receive funding to implement multicenter trials. To date, 37 TIN consultations have resulted in multicenter trials that receive TIN operational assistance with study startup and/or enrollment/retention.

The TIN maintains a publicly accessible website where trial-related materials and webinars are available to research communities outside of the CTSA network. Publicly available materials include a toolbox of trial-related resources, selected publications about trial support and conduct, and an archive of training events.

**Figure 4.**
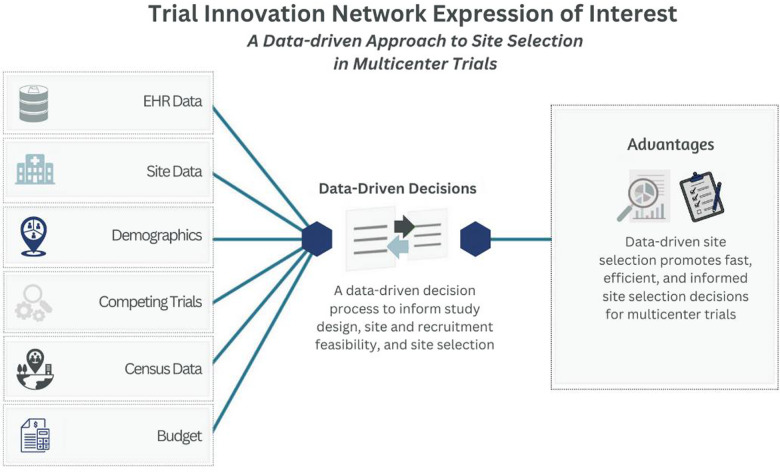
Through the TIN Expression of Interest (EOI) investigative teams can leverage a data-driven approach to inform study design and assist with site selection.

Some materials and resources are limited to CTSAs and their affiliates or to researchers who collaborate or partner with the TIN. One TIN resource that collaborating investigative teams can leverage is the EHR-based cohort assessment process developed for RIC consultations (Fig. 4). Using a data-driven approach to center selection for multicenter trials, the RIC applies a low burden, low tech, high-yield discovery model to retrieve study population estimates from electronic health record (EHR) systems across interested CTSA Program hub centers and their affiliates. This fast and flexible method informs study design decisions, center feasibility, and center selection [16]. Other collaborating resources include protocol and trial analysis designs, participant recruitment and retention suggestions, single IRB support, and multicenter engagement strategies [13].

The national CTSA Consortium provides a unique opportunity for multicenter trialists to partner with CTSA hubs. The TICs and RIC collaborate to send multicenter trial announcements to hubs for circulation among investigative teams that might be interested in potential trial opportunities. This process is called Expression of Interest (EOI) and involves presenting trial details to hub points of contact and potential site PIs who can then assess if a trial is feasible and of interest to their clinical practices. In the spirit of informativeness, the EOI process includes a local electronic health record (EHR)-based cohort assessment [16] and an online seminar where hub teams can ask questions about a particular study. The EOI process often includes sharing of the protocol and budget, participant selection criteria, and details of the intervention for hub investigative teams to provide comments. The EOI is an important informative mechanism by which the TIN can support investigators transitioning a single center trial to a multicenter trial. The EOI process has been used in more than 55 studies, including 13 studies that were moving from a single center to a multicenter study design.

## Principle 3: Carefully Consider Budget and Contract Factors That May Affect Informativeness

Investigative teams may not carefully and realistically consider study budgets, and center contracts may overlook regulatory requirements or underestimate costs, all of which could lead to problems with trial feasibility.

### Informed Approach

*When engaging centers, sharing the center budget is as important as sharing the protocol and the science. Budgets are best developed when participating centers confirm that cost estimates match expenses within their own institutions.*

### Description

Typically, a multicenter study budget is broken into central costs incurred by the trial sponsor and subcontractor costs that cover reimbursements to multiple enrolling centers and vendors acting as suppliers or performing outsourced services. Overall, the costliest factors across all trial stages are clinical procedure (patient care) costs (15%-22%), retention costs at enrolling centers (9%-16%), and operational staff costs (11%-29%), quality assurance (QA) monitoring costs (9%-14%), and core laboratory costs (4%-12%) borne by coordinating centers [1].

Too often, trial estimates do not hold up to actual expenditures, and enrollment center budgets are often overlooked as causes of expenditure underestimations. Costly mistakes include failing to survey centers for average patient care costs, underestimating screening and recruitment requirements, budgeting personnel at too low effort or salary, underestimating how sIRB costs are multiplied by the number of relying centers, and budgeting future vendor costs based on past experiences without procuring actual bids. As stated earlier, use of new or unfamiliar technologies that may require additional internal and/or external review and approval can also take time and incur cost. Other overlooked costs include staffing turnover, database updates, IRB amendments, and foremost, delayed timelines. Recently, cost burdens have been attributed to IRB reviews, whether with one protocol reviewed by multiple IRBs or with a single IRB. Changes and amendments while a study is underway can incur substantial costs. As an example, the median direct cost to implement a substantial amendment for Phase II protocols is $141,000 and for Phase III protocols is $535,000 [17]. The administrative burden of balancing and justifying budgets is heightened by all these accounting shortfalls.

Even for trials where the budget is on target, line-item expenses are rarely static and require constant attention and updates as issues are identified. Throughout the trial life cycle, continuous monthly trial spending reviews and reforecasting are important, as actual expended and obligated resources are tracked and compared to the trial’s business plan.

### Principle 3 at Work in the Trial Innovation Network

Delays in study start-up and completion, and the resulting cost burdens, have been attributed in part to the lack of user-friendly budgeting support and the absence of center contract and budget standardization. To support multicenter study investigators, the TIN developed a tool for center budgeting, providing comparisons of patient costs to reimbursement budgets to help inform participating centers and sponsors as to whether proposed budgets are reasonable [18]. With CTSA hub staff as collaborators, the TIN also helps investigators assess financial feasibility, using EHR-derived cohorts to understand local and regional catchment populations to project recruitment and reimbursements, and by sharing budgets early with potential multicenter investigators. Another resource provided by the TIN is a downloadable subaward template for Federally funded clinical trials that most U.S. institutions can accept, negating the need for tedious negotiations over contracts. The TIN has helped multicenter investigators with budgeting and contracts at 73 centers for 9 studies.

## Conclusion

Clinical trials are an important part of the translational science spectrum. Single center studies may be advantageous, especially when testing a new research hypothesis in a small population. For small-scale studies, a single center can be more homogeneous in equipoise, training, and management. A single center can also be more efficient: funding needs are smaller and can be easier to obtain, the study will be easier to conduct, and IRB approvals can be secured more quickly [19].

When it becomes important to generalize findings in more diverse populations and settings, larger multicenter trials come into play. Multicenter trials have many benefits, such as a larger number of participants, different geographic locations, inclusion of a wider range of population groups, and the ability to compare results among centers, which will increase the overall generalizability and informativeness of the trial results. However, multicenter trials require more resources and more diverse training and management than single center studies. Inefficient and ineffective processes in multicenter trials can lead to uninformative results. When setting up a multicenter trial, early considerations include determining the timelines and costs for developing the critical processes associated with receiving and analyzing study data, providing logistical support to physically distant colleagues and personnel at multiple institutions, and documenting operating procedures to ensure a systematic approach to all trial procedures. Diverse teams with complementary expertise leveraging existing processes and systems can shorten timelines and hold down costs.

The NIH recognizes the importance of multicenter trial planning time and factors this into the structure of awards for multicenter trials. Planning time for multicenter trials is needed so that investigative teams can engage collaborators, obtain regulatory approvals, and train center staff. The TIN recognizes that many multicenter investigative teams may not know how to efficiently and effectively plan for all the challenges associated with multicenter studies. The TIN has therefore developed many resources to help investigative teams learn how to track their planning period progress, prioritize tasks, and use reasonable time estimates when setting timelines and milestones.

The TIN has published extensively about its work, including barriers and roadblocks in clinical trial operations [20]. Many TIN resources are publicly available via the online TIN Toolbox, and training events are also advertised on the TIN website, with previous events archived for ongoing access. Trial investigators seeking a formal TIN consultation can collaborate with their local CTSA that will review and provide local resources or support a submission to the TIN portal. CTSA liaison teams are listed on the TIN website to help investigators connect with local resources and support.

The breadth and complexity of multicenter studies can stress resources and will challenge the skillsets of even very experienced multicenter trialists. To have the best chance of conducting an informative multicenter trial, every PI, regardless of experience, must assemble early a diverse team and empower the team to plan and to confer with colleagues, funding officers, and CTSA staff to jointly define solutions before there are problems, and to develop a realistic budget that matches the details and needs of the trial. The CTSA Program hubs and the TIN are resources that can help investigative teams initiate multicenter trials, the cornerstone of informative, evidence-based medicine.
